# Effect of the Use of Different Acoustic Stimuli on Cortical Auditory Evoked Potentials and Autonomic Cardiac Modulation

**DOI:** 10.1155/2018/5171304

**Published:** 2018-06-03

**Authors:** Simone Fiuza Regaçone, Vitor Engrácia Valenti, Ana Cláudia Figueiredo Frizzo

**Affiliations:** Department of Speech Therapy and Audiology, Faculty of Philosophy and Sciences, UNESP, Avenida Hygino Muzzi Filho 737, 17525-900 Marília, SP, Brazil

## Abstract

**Purpose:**

The aim of this study was to analyze the relationship between Cortical Auditory Evoked Potentials (CAEPs) measurements and autonomic cardiac modulation in relation to different acoustic stimuli and to verify which of these stimuli have more influence on the autonomic nervous system.

**Methods:**

Sixty healthy women, aged between 18 and 25 years, participated in this study. Prior to the CAEP examination, blood pressure and resting heart rate were measured using a stethoscope, sphygmomanometer, and the Polar RS800CX cardiofrequency measures. After the collection of these measures, the CAEP test was started simultaneously with the HRV collection.

**Results:**

All the HRV indices presented correlations with the components of the CAEPs. During the acoustic stimulation, a predominance of the modulation of the autonomic parasympathetic nervous system was observed. The harmonic and disharmonic stimuli were the ones that presented the most correlations between the measures analyzed in this study.

**Conclusions:**

There was an association between CAEP and cardiac autonomic modulation in relation to different acoustic stimuli. Among the acoustic stimuli used in this study, the ones that most influenced the autonomic cardiac modulation were harmonic and disharmonic stimuli, which are acoustically more complex stimuli.

## 1. Introduction

In the course of its history, audiology has gained strength and evolved, along with technological progress; advanced methods in electrophysiology such as Cortical Auditory Evoked Potential (CAEP) have been used in order to unravel how the process of information occurs in the central nervous system [1, 2].

In this sense, CAEPs have become essential since this technique is noninvasive and can be used in all age groups, in addition to their contributions to the study of normal and altered auditory cortical functioning [2].

Another method of fundamental importance in the scientific environment has been the analysis of Heart Rate Variability (HRV), a widely disseminated tool in the literature capable of evaluating cardiac autonomic modulation, which due to its importance and complexity mobilized the European and American Society of Cardiology to form a task force [3] in order to standardize this analysis.

Although these two methods differ in the area of knowledge, they are believed to be related, given some studies [4–7] that address this issue. However, this theme still needs to be further studied in the scientific environment, due to its complexity and also because there is a restricted literature.

Lawrence and Barry [4, 5] correlated cardiac evoked potentials with event-related potentials and showed that there is a link between the central nervous system and autonomous nervous system in reflecting on some aspects of auditory stimulus processing.

In a study with event-related auditory evoked potentials and HRV indices during the simulated conduction test of mental fatigue, a significant reduction in P300 amplitude was observed, indicating that when the individual was in induced mental fatigue there was a decrease in attention for the auditory task [6].

A recent study of healthy young women relating CAEP and cardiac parameters showed that during the acoustic stimulation, when the electrical activity reaches the cortex associating the initials attention and decoding of the stimulus, it is processed in the auditory pathway through the alert activity, and thus the heart rate values tend to increase and the amplitudes of N1 and P3a decrease [7].

Complementary studies in this direction will contribute to the understanding of different physiological responses in the human organism, such as blood pressure and heart rate in face of auditory stimuli.

Therefore, the aim of this study was to analyze the association between CAEP and cardiac autonomic modulation in relation to different acoustic stimuli and to verify which one has more influence on cardiac autonomic modulation.

## 2. Methods

### 2.1. Ethics

The study received ethics approval from the University Brazilian Public Human Participants Ethics Research Committee (Reference 419/2012).

### 2.2. Participants

Sixty healthy women aged 18 to 25 years participated in this study. Participants were divided into three groups, consisting of 20 healthy women each. Each group received a type of auditory stimulus: Group I (frequency stimulus), Group II (harmonic stimulus), and Group III (disharmonic stimulus).

For the composition of the groups studied, the following inclusion criteria were established: female gender; age range between 18 and 25 years; absence of complaints or current history of affections of the auditory system; absence of cardiorespiratory, cognitive, psychiatric, neurological, and other known disorders that prevent the subject from performing the procedures, as well as treatment with medications that influence cardiac autonomic regulation; individuals who have not ingested alcoholic beverages within the last 24 hours prior to the examination and who have stopped smoking for at least one year; individuals not in the menstrual period.

### 2.3. Initial Procedure

After the selection of the sample, all the participants of the research received a free and informed consent term.

As an initial procedure audiological anamnesis was performed aiming to investigate the history of general and auditory health of the volunteers. Afterward, the external auditory meatus was inspected to verify if there was excess cerumen.

Audiological exams were performed, such as the investigation of tonal audiometry in the frequencies from 250 to 8000 Hz in the airway. Tympanometry was also performed, and acoustic stapedial reflexes were verified in the frequencies from 500 to 4000 Hz.

The normality of the peripheral auditory system was confirmed by thresholds less than or equal to 25 dBNA [8], type A tympanogram, the indication of normal mobility of the tympanoossicular system [9], and when ipsilateral and contralateral reflexes were present.

The volunteers were instructed not to drink alcohol and caffeine in the 24 hours before the evaluation. The collection was performed individually, between 12 and 15 hours to standardize interferences of the circadian rhythm, and the volunteers were instructed to remain at rest, avoiding conversations during the collection.

BP was measured using the stethoscope and sphygmomanometer. The Polar RS800CX (Polar Electro, Finland) heart rate receiver was then placed on the thorax of the participants, in the distal third of the sternum. The participants were then placed in an armchair and remained seated for 10 minutes for basal HR measurement.

After BP and HR were obtained, CAEP and HRV were performed simultaneously in the acoustically treated and temperature controlled, at 24°C, room.

### 2.4. Electrophysiology

For CAEP analysis, electrodes with microporous adhesive tape were fixed after cleaning the skin with abrasive paste, and electrolytic paste was used to improve the electrical conductivity. The impedance of each electrode did not exceed 5 Kohms and did not exceed 2 Kohms between the impedance of the electrodes [2]. The active electrodes (+) were attached in* Cz* and* Fz*, the reference electrodes (−) were attached in the right ear lobe* (A2)* and left ear lobe* (A1)*, and the ground electrode was positioned on the forehead.

Participants were accommodated in a reclining chair and oriented to remain relaxed with their eyes open and alert, watch a video (without sound) to distract themselves, and not direct their attention to the stimulus that was presented in an oddball paradigm (rare stimulus presented randomly amongst frequent stimuli).

Three different auditory stimuli were used to investigate the CAEP components: frequency (CAEPf), harmonic (CAEPhar), and disharmonic (CAEPdishar).

For frequency sweeping** (CAEPf)**, a frequent stimulus of 100 ms was used, at the 1000 Hz frequency, with tone burst type with 20 ms (rise), 60 ms (plateau), and 20 ms (fall), as well as the rare stimulus of 100 ms at the frequency of 2000 Hz, with type tone burst with 20 ms (rise), 60 ms (plateau), and 20 ms (fall).

**Figure 1:**
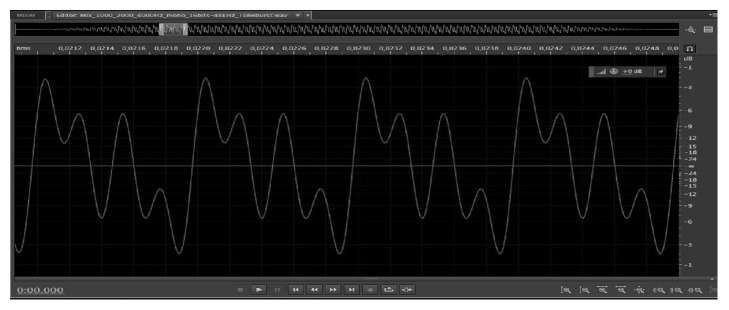
Screen capture of the harmonic stimuli.

The frequent stimulus used in harmonic scanning** (CAEPhar)** was a 96 ms low frequency harmonic set consisting of 250, 500, and 1000 Hz, with tone burst with 6 cycles (rise), 12 cycles (plateau), and 6 cycles (fall) (24 ms, 48 ms, and 24 ms), and the rare stimulus was a 96 ms high frequency harmonic set consisting of 1000, 2000, and 4000 Hz, with tone burst with 24 cycles (rise), 48 cycles (plateau), and 24 cycles (fall), that is, 24 ms, 48 ms, and 24 ms (Figure 1).

**Figure 2:**
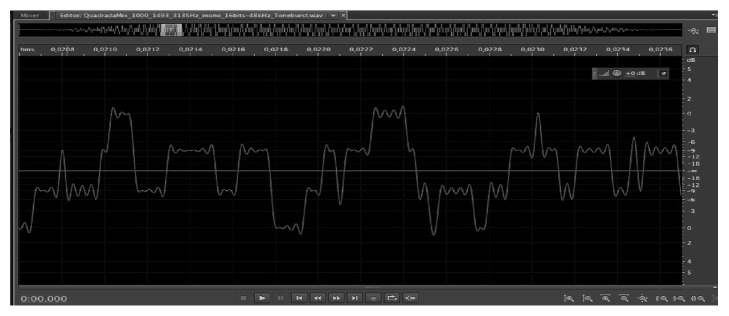
Screen capture of the disharmonic stimuli.

In the disharmonic sweep** (CAEPdishar)**, low frequency harmonic set of 96 ms was used for the frequent stimulus consisting of 250, 500, and 1000 Hz, with tone burst with 6 cycles (rise), 12 cycles (plateau), and 6 cycles (fall), 24 ms, 48 ms, and 24 ms, and the rare stimulus was a 96 ms high frequency disharmonic set consisting of 1000, 1493, and 3135 Hz, with tone burst with 24 cycles (rise), 48 cycles (plateau), and 24 cycles (fall), that is, 24 ms, 48 ms, and 24 ms (Figure 2).

The stimuli were presented randomly in the proportion of 20% of rare stimuli of a total of 300 stimuli recorded in a window of 500 ms, with sensitivity of 100 microvolts, alternating polarity—with band pass filtering of 0.5–30 Hz—monaural stimuli, and stimulation rate of 1.1 stimuli/second in the intensity of 70 dB NA [2].

For the final analysis of the results, the records obtained in Cz were used, because they showed the best wave morphology.

The latency and amplitude values of the components N1, P2, and N2 were marked following criteria established in the literature. This complex was identified in the appearance of the first three waves, in the highest peak, in sequence in the negative-positive-negative polarities, respectively, between 60 and 300 ms [10].

As for the P3a component, its latency was marked between 220 and 350 ms [11]. For the identification of the MMN component, the largest negative polarity wave was considered, between the values of latency from 100 to 300 ms, visualized in the subtraction of the tracing of the rare stimulus to the tracing of the frequent stimulus [12, 13].

The examinations lasted approximately 50 minutes. As a standard, to maintain a quality examination, when the volunteers presented myogenic interference, position changes were suggested and when necessary the examination was repeated.

### 2.5. Heart Rate Variability

In order to investigate the HRV, the Polar RS800CX receptor allowed HR beat to be recorded throughout the experimental protocol with a sampling rate of 1000 Hz. A range of five minutes was selected from the period of greatest stability of the signal, and the only series with more than 256 intervals (heart rate interval) were used for analysis [3].

To eliminate premature ectopic beats and artifacts, digital and manual filtration were performed, and only series with more than 95% of sinus beats were included in this study [14].

Cardiac autonomic modulation was quantified using time domain and frequency domain analysis.

Time domain analysis was performed using mean HR (heart rate), SDNN (standard deviation of mean RR intervals), RMSSD (root mean square of difference between adjacent normal RR intervals in a time interval), and pNN50 (percentage of adjacent RR intervals with duration difference greater than 50 ms).

For the HRV analysis in the frequency domain, low frequency (LF: 0.04–0.15 Hz) and high frequency (HF: 0.15–0.40 Hz) spectral components were used, in ms^2^. The spectral analysis was calculated using the Fast Fourier Transform algorithm.

Linear indexes in the time and frequency domain were analyzed using Kubios HRV analysis software version 2.1 [15].

### 2.6. Statistical Analysis

Data normality was determined using the Shapiro-Wilk test to compare the results among individuals from the same group. To verify the correlation between the variables, the Pearson correlation test was applied to parametric distributions. The Spearman correlation test was applied for nonparametric distributions. Strong correlation values were considered for *r* > 0.5, moderate correlation was considered for “*r*” values between 0.3 and 0.49, and weak correlation was considered for “*r*” values < 0.3. Test differences were considered statistically significant when the “*p*” value was less than or equal to 0.05 (5%). The statistical software used was BioStat 2009 Professional 5.8.4®.

## 3. Results

As shown in Table 1, there was correlation between the CAEP components and the HR.

- Harmonic stimulus: strong positive correlation of the HR with the N2 latency in the right ear; strong negative correlation with the P3a amplitude in the right ear and with the P2 latency in the left ear.
- Disharmonic stimulus: strong positive correlation with the N1 and N2 latency in the left ear; strong negative correlation with the N1 latency in the right ear and the P3a latency in the left ear.

Table 2 shows correlation between the CAEP components and the RMSSD index of the HRV.

- Frequency stimulus: strong positive correlation in the P3a amplitude in the left ear.
- Harmonic stimulus: strong positive correlation of the amplitudes of P3a and MMN in the right ear; a moderate negative correlation in the P2 latency in the left ear and in the N2 latency in the right ear.
- Disharmonic stimulus: moderate negative correlation of the latency of N1 in the left ear and a moderate positive correlation in the P3a amplitude in the right ear.

As shown in Table 3, there was correlation between the components of the CAEP and the SDNN index of the HRV.

- Harmonic stimulus: a moderate negative correlation of the latency of P2 in the left ear; a moderate positive correlation of the amplitude of P3a in the right ear.
- Disharmonic stimulus: a strong negative correlation of the latency of P3a in the left ear and a moderate positive correlation of the latency of P3a in the right ear.

Table 4 presents the correlation values between the CAEP components and the pNN50 index of the HRV.

- Frequency stimulus: strong positive correlation of the P3a amplitude in the left ear.
- Harmonic stimulus: strong negative correlation of the latency of N2 in the right ear and in the latency of P2 in the left ear and strong positive correlation of the P3a and MMN amplitude in the right ear.
- Disharmonic stimulus: strong negative correlation in the latency of N1 in the left ear and moderate positive correlation in the amplitude of P3a in the right ear.

Table 5 shows correlation between the CAEP components and the LF index (ms^2^) of the HRV.

  Frequency stimulus: moderate positive correlation of N1 latency in the right ear and a moderate negative correlation in the N1 amplitude in the left ear.

As shown in Table 6, there was correlation between the HF index (ms^2^) and the components of the CAEP.

- Frequency stimulus: strong positive correlation in the amplitude of P3a in the left ear and a moderate positive correlation in the right ear.
- Harmonic stimulus: moderate positive correlation in the amplitude of P3a in the right ear.
- Disharmonic stimulus: strong positive correlation in the latency of P3a in the right ear.

## 4. Discussion

The results of the present study showed a correlation between the indexes of the HRV, in time and frequency domain with CAEP components. These findings confirm other studies [4–7].

The generation of components of auditory cortical potentials, from the earliest to the late components, occurs in a procedural way, one of which is a prerequisite for the generation of the other. They become more elaborate as they travel through the auditory pathway, beginning in the upper olive groves; they depend on several other neural circuits along the ascending projections, through the lateral lemniscus, arriving at the inferior colliculus which receives binaural afferents, preserving the tonotopic organization complex [16].

From the anatomical and physiological perspective, the relationship between the auditory and cardiovascular systems is evident. The reticular formation, which occupies the entire central region of the brainstem, from the bulb to the midbrain, is one of the structures connected to the two systems, responsible for the regulation of alertness, subsidizes the attentional process, and controls the autonomic nervous system, rhythm, and blood pressure [17].

During the auditory stimulation with the harmonic and disharmonic stimuli, there was a predominance of a strong positive correlation between HR and all the components of CAEP, with the exception of MMN.

The auditory component MMN presented a strong positive correlation with HRV indices in the time domain (RMSSD and pNN50) only in the harmonic stimulus in right ear. Such a component seems to be more associated with the processes related to the distinction of the acoustic characteristics that require more participation of the parasympathetic nervous system.

In the analysis of spectral frequency indices, correlation was found between the LF index (ms^2^) and the N1 component. The HF index (ms^2^) presented a correlation with the auditory component P3a. These findings show us the interaction between the sympathetic and vagal stimulation during auditory sensory evaluation.

Most of HRV measures' analyses used in this study bring about information about the parasympathetic nervous system, which suggests that throughout the auditory stimulation the system eventually adapts probably due to the repetition of the stimulus.

It is believed that the autonomic nervous system turns off the alert system and the system is restored since this repetitive stimulus does not pose risks to the body. In addition, when the assessed subject is on alert status, the heart rate pattern tends to stabilize [18, 19].

A recent study of healthy young women relating CAEP and cardiac parameters showed that during auditory stimulation HR values tend to increase and the amplitudes of N1 and P3a decrease [7]. In contrast, the results of this research presented a relationship not only with the components N1 and P3a but with all the components of the CAEP as a function of the use of complex stimuli.

In relation to the other approach of this study, on the different acoustic stimulus and effects in the systems, it can be stated that the harmonic and disharmonic stimuli promoted a broader activation of the auditory structures because they presented different frequency spectra and required a larger tonotopic organization [20].

It is believed that these stimuli tend to a greater excitation of the whole system due to their complexity since they are composed of six different frequency bands between bass and treble, unlike the frequency stimulus that is composed only by the frequencies 1 kHz and 2 kHz. Discrimination of the stimuli, harmonic and disharmonic, requires more of the subject because they present more acoustic characteristics and stimulate the cochlea as a whole, and in contrast, the frequency stimulus stimulates a more restricted area of the cochlea and central nervous system.

Thus, the harmonic and disharmonic stimuli require more of the auditory system which justifies our results with greater statistical significance in these stimuli when compared to the frequency stimuli. These stimuli promoted greater activation not only of the auditory system but also of the autonomic nervous system.

It is worth mentioning that both the exogenous components, N1 and P2, and the endogenous components, N2, P3a, and MMN of the CAEP, showed a correlation with the HRV indices, which indicates an action of the autonomic cardiac modulation on the generation of these auditory components since the decoding of the initial auditory stimulus, the early cortical processing of sound and characteristics, and automatic perception, discrimination, and recognition of sounds until the alert activity during the initial assignment of attention or redirection of sensory attention triggered by distracting stimuli that occur as a functional effect of the use of the oddball paradigm and the stimulus unpredictability [1, 2].

Finally, in this study, it was possible to observe the interaction between the auditory system and the cardiovascular system based on the analysis of the measures presented here. During the processing of auditory information, the autonomic nervous system participates in the modulation of cardiovascular functions and also of the auditory responses through the action of the sympathetic and parasympathetic systems.

## 5. Conclusion 

Therefore, it is concluded that there is an association between CAEP and autonomic cardiac modulation for different acoustic stimuli and that the autonomic nervous system predominance was parasympathetic. Among the acoustic stimuli used in this study, those that most influenced the autonomic modulation were the harmonic and disharmonic, which are more complex stimuli.

This study has provided new insights into the interplay between the central and autonomic nervous systems; these data offer support for our better understanding of the interference of acoustic stimuli in the heart rate modulation.

## Figures and Tables

**Table 1:** Correlation between the components of the CAEP and the HR.

Ear	Components	Stimulus					
		Frequency		Harmonic		Disharmonic	
		*r*	*p*	*r*	*p*	*r*	*p*
RE	Lat N1	−0,04	0,84	−0,17	0,45	−**0,50**	**0,02** ^*∗*^
	Lat P2	0,35	0,12	0,31	0,17	0,12	0,59
	Lat N2	0,36	0,11	**0,55**	**0,01** ^*∗∗*^	0,18	0,42
	Lat P3a	0,21	0,35	0,38	0,09	0,01	0,93
	Lat MMN	0,40	0,07	0,34	0,13	0,01	0,94
	Amp N1	−0,25	0,28	0,32	0,16	0,07	0,74
	Amp P2	0,17	0,46	0,39	0,08	0,37	0,10
	Amp N2	0,08	0,73	−0,20	0,37	0,06	0,77
	Amp P3a	−0,03	0,86	−**0,54**	**0,01** ^*∗*^	−0,27	0,24
	Amp MMN	0,27	0,24	−0,37	0,10	0,15	0,51

LE	Lat N1	−0,24	0,28	0,04	0,83	**0,57**	**0,00** ^*∗*^
	Lat P2	0,16	0,47	**0,43**	**0,05** ^*∗*^	0,24	0,30
	Lat N2	0,18	0,42	0,22	0,34	**0,50**	**0,02** ^**∗∗**^
	Lat P3a	0,12	0,59	0,39	0,08	**0,44**	**0,05** ^*∗*^
	Lat MMN	0,14	0,55	0,17	0,46	0,16	0,49
	Amp N1	−0,26	0,25	0,10	0,66	0,16	0,49
	Amp P2	0,07	0,74	0,02	0,92	0,11	0,64
	Amp N2	−0,07	0,74	−0,05	0,81	−0,06	0,77
	Amp P3a	−0,35	0,12	−0,33	0,15	−0,02	0,90
	Amp MMN	0,02	0,90	−0,19	0,41	0,11	0,64

RE: right ear; LE: left ear; Lat: latency; Amp: amplitude; value of *p* ≤ 0.05, ^*∗*^Pearson correlation; ^*∗∗*^Spearman correlation; CAEP: Cortical Auditory Evoked Potential; HR: heart rate.

**Table 2:** Correlation between the components of the CAEP and the RMSSD index.

Ear	Components	Stimulus					
		Frequency		Harmonic		Disharmonic	
		*r*	*p*	*r*	*p*	*r*	*p*
RE	Lat N1	0,22	0,34	0,23	0,32	0,40	0,07
	Lat P2	−0,23	0,31	−0,36	0,11	0,06	0,79
	Lat N2	−0,26	0,26	−**0,49**	**0,02** ^*∗∗*^	0,19	0,41
	Lat P3a	−0,04	0,85	−0,25	0,28	0,38	0,09
	Lat MMN	−0,31	0,17	−0,34	0,13	0,10	0,65
	Amp N1	0,13	0,55	−0,29	0,20	−0,02	0,91
	Amp P2	−0,26	0,25	−0,22	0,33	−0,34	0,13
	Amp N2	−0,16	0,48	0,14	0,53	−0,18	0,44
	Amp P3a	0,40	0,07	**0,56**	**0,00** ^*∗∗*^	**0,44**	**0,04** ^*∗∗*^
	Amp MMN	−0,31	0,17	**0,51**	**0,02** ^*∗∗*^	−0,07	0,75

LE	Lat N1	0,23	0,31	−0,24	0,30	−**0,46**	**0,03** ^*∗∗*^
	Lat P2	0,05	0,81	−**0,45**	**0,04** ^*∗∗*^	−0,26	0,25
	Lat N2	−0,02	0,92	−0,37	0,10	−0,36	0,11
	Lat P3a	0,06	0,78	−0,29	0,20	−0,29	0,20
	Lat MMN	−0,02	0,90	−0,30	0,19	−0,04	0,85
	Amp N1	0,31	0,17	0,06	0,79	−0,29	0,20
	Amp P2	−0,23	0,31	0,03	0,86	−0,24	0,29
	Amp N2	−0,14	0,53	−0,00	0,98	−0,04	0,84
	Amp P3a	**0,51**	**0,01** ^*∗∗*^	0,30	0,19	0,27	0,24
	Amp MMN	−0,09	0,69	0,21	0,37	−0,21	0,35

RE: right ear; LE: left ear; Lat: latency; Amp: amplitude; value of *p* ≤ 0.05, ^*∗*^Pearson correlation; ^*∗∗*^Spearman correlation; CAEP: Cortical Auditory Evoked Potential; RMSSD: square root of the sum of successive differences between normal RR intervals adjacent to the square.

**Table 3:** Correlation between the components of CAEP and the SDNN index.

Ear	Components	Stimulus					
		Frequency		Harmonic		Disharmonic	
		*r*	*p*	*r*	*p*	*r*	*p*
RE	Lat N1	0,37	0,10	0,04	0,83	0,25	0,28
	Lat P2	−0,31	0,18	−0,33	0,14	0,20	0,38
	Lat N2	−0,32	0,16	−0,41	0,06	0,42	0,06
	Lat P3a	−0,20	0,37	−0,34	0,14	**0,49**	**0,02** ^*∗∗*^
	Lat MMN	−0,42	0,06	−0,26	0,26	0,16	0,48
	Amp N1	0,07	0,75	−0,08	0,73	−0,03	0,89
	Amp P2	−0,26	0,26	−0,14	0,54	−0,07	0,76
	Amp N2	0,01	0,95	0,35	0,12	0,09	0,69
	Amp P3a	0,39	0,08	**0,45**	**0,04** ^*∗*^	0,06	0,79
	Amp MMN	−0,24	0,29	0,31	0,17	0,02	0,91

LE	Lat N1	−0,05	0,82	0,05	0,82	−0,32	0,15
	Lat P2	0,19	0,41	−**0,45**	**0,04** ^*∗∗*^	−0,22	0,35
	Lat N2	0,13	0,57	−0,21	0,37	−0,39	0,08
	Lat P3a	0,11	0,64	0,10	0,64	−**0,57**	**0,00** ^*∗∗*^
	Lat MMN	0,09	0,69	−0,18	0,42	−0,20	0,39
	Amp N1	0,04	0,86	0,20	0,38	−0,33	0,15
	Amp P2	−0,07	0,74	−0,26	0,25	−0,23	0,32
	Amp N2	−0,22	0,34	0,23	0,32	−0,05	0,82
	Amp P3a	0,29	0,20	0,16	0,48	0,08	0,71
	Amp MMN	0,00	0,97	0,15	0,50	−0,28	0,22

RE: right ear; LE: left ear; Lat: latency; Amp: amplitude; value of *p* ≤ 0.05, ^*∗*^Pearson correlation; ^*∗∗*^Spearman correlation; CAEP: Cortical Auditory Evoked Potential; SDNN: standard deviation of all normal RR intervals.

**Table 4:** Correlation between the components of CAEP and the pNN50 index.

Ear	Components	Stimulus					
		Frequency		Harmonic		Disharmonic	
		*r*	*p*	*r*	*p*	*r*	*p*
RE	Lat N1	0,03	0,89	0,19	0,40	0,37	0,10
	Lat P2	−0,18	0,43	−0,40	0,07	0,00	0,97
	Lat N2	−0,21	0,36	−**0,51**	**0,02** ^*∗∗*^	0,20	0,38
	Lat P3a	−0,00	0,98	−0,25	0,27	0,39	0,08
	Lat MMN	−0,35	0,12	−0,37	0,10	0,11	0,63
	Amp N1	0,18	0,43	−0,32	0,16	−0,03	0,87
	Amp P2	−0,21	0,35	−0,25	0,27	−0,30	0,18
	Amp N2	−0,15	0,52	0,13	0,58	−0,18	0,44
	Amp P3a	0,41	0,07	**0,60**	**0,00** ^*∗∗*^	**0,42**	**0,05** ^*∗∗*^
	Amp MMN	−0,37	0,10	**0,50**	**0,02** ^*∗∗*^	−0,13	0,57

LE	Lat N1	0,16	0,48	−0,20	0,37	−**0,50**	**0,02** ^*∗∗*^
	Lat P2	0,07	0,75	−**0,52**	**0,01** ^*∗*^	−0,30	0,18
	Lat N2	0,01	0,93	−0,41	0,07	−0,39	0,08
	Lat P3a	0,12	0,59	−0,21	0,36	−0,34	0,13
	Lat MMN	0,00	0,99	−*0,44*	*0,04* ^*∗*^	−0,13	0,58
	Amp N1	0,37	0,10	0,01	0,93	−0,23	0,31
	Amp P2	−0,23	0,32	−0,05	0,82	−0,19	0,40
	Amp N2	−0,21	0,36	−0,02	0,90	−0,06	0,80
	Amp P3a	**0,57**	**0,00** ^*∗∗*^	0,21	0,35	0,26	0,26
	Amp MMN	−0,02	0,91	0,11	0,64	−0,28	0,22

RE: right ear; LE: left ear; Lat: latency; Amp: amplitude; value of *p* ≤ 0.05, ^*∗*^Pearson correlation; ^*∗∗*^Spearman correlation; CAEP: Cortical Auditory Evoked Potential; pNN50: percentage of normal RR intervals that differ by more than 50 milliseconds from their adjacent ones.

**Table 5:** Correlation between the components of CAEP and the LF index (ms^2^).

Ear	Components	Stimulus					
		Frequency		Harmonic		Disharmonic	
		*r*	*p*	*r*	*p*	*r*	*p*
RE	Lat N1	**0,45**	**0,04** ^*∗*^	−0,14	0,54	−0,06	0,79
	Lat P2	−0,16	0,48	−0,07	0,76	0,19	0,41
	Lat N2	−0,29	0,20	−0,24	0,29	0,19	0,40
	Lat P3a	−0,29	0,20	−0,31	0,18	0,18	0,44
	Lat MMN	−0,30	0,19	−0,04	0,83	−0,08	0,70
	Amp N1	−0,02	0,91	0,07	0,76	0,17	0,46
	Amp P2	−0,37	0,09	−0,04	0,86	0,08	0,73
	Amp N2	0,04	0,84	0,31	0,17	0,22	0,34
	Amp P3a	0,38	0,09	0,04	0,85	−0,07	0,76
	Amp MMN	−0,18	0,42	0,22	0,33	0,12	0,59

LE	Lat N1	−0,22	0,33	−0,04	0,84	−0,11	0,62
	Lat P2	−0,22	0,33	−0,06	0,79	0,00	0,96
	Lat N2	−0,01	0,95	−0,02	0,91	−0,02	0,92
	Lat P3a	0,02	0,93	−0,11	0,62	−0,16	0,49
	Lat MMN	−0,08	0,72	−0,17	0,46	−0,07	0,75
	Amp N1	−**0,44**	**0,04** ^*∗∗*^	0,16	0,49	−0,04	0,83
	Amp P2	0,04	0,84	−0,01	0,94	−0,1	0,67
	Amp N2	−0,09	0,69	0,24	0,29	−0,01	0,96
	Amp P3a	0,04	0,86	0,17	0,46	0,09	0,69
	Amp MMN	−0,18	0,44	0,03	0,88	−0,25	0,27

RE: right ear; LE: left ear; Lat: latency; Amp: amplitude; value of *p* ≤ 0.05, ^*∗*^Pearson correlation; ^*∗∗*^Spearman correlation; CAEP: Cortical Auditory Evoked Potential; LF: low frequency (0,04 to 0,15).

**Table 6:** Correlation between the components of CAEP and the HF index (ms^2^).

Ear	Components	Stimulus					
		Frequency		Harmonic		Disharmonic	
		*r*	*p*	*r*	*p*	*r*	*p*
RE	Lat N1	0,15	0,52	0,13	0,58	0,20	0,38
	Lat P2	−0,17	0,46	−0,23	0,31	0,07	0,75
	Lat N2	−0,22	0,34	−0,29	0,21	0,32	0,16
	Lat P3a	−0,05	0,80	0,13	0,56	**0,55**	**0,01** ^*∗∗*^
	Lat MMN	−0,37	0,10	−0,15	0,51	0,15	0,51
	Amp N1	0,07	0,73	−0,21	0,35	0,09	0,68
	Amp P2	−0,05	0,81	−0,04	0,83	−0,29	0,21
	Amp N2	0,03	0,88	0,07	0,75	0,04	0,85
	Amp P3a	**0,49**	**0,02** ^*∗∗*^	**0,47**	**0,03** ^*∗∗*^	0,11	0,61
	Amp MMN	−0,13	0,58	0,14	0,53	0,07	0,74

LE	Lat N1	0,07	0,76	0,18	0,43	−0,09	0,67
	Lat P2	0,24	0,30	−0,24	0,29	0,11	0,61
	Lat N2	0,21	0,35	−0,40	0,07	0,00	0,96
	Lat P3a	0,12	0,60	0,21	0,36	−0,02	0,90
	Lat MMN	0,16	0,49	−0,10	0,65	0,15	0,50
	Amp N1	0,15	0,50	0,06	0,78	−0,29	0,21
	Amp P2	−0,06	0,80	−0,18	0,42	−0,08	0,72
	Amp N2	−0,17	0,44	0,04	0,84	0,23	0,31
	Amp P3a	**0,53**	**0,01** ^*∗∗*^	0,29	0,20	−0,05	0,83
	Amp MMN	0,00	0,99	−0,06	0,78	−0,15	0,50

RE: right ear; LE: left ear; Lat: latency; Amp: amplitude; value of *p* ≤ 0.05, ^*∗*^Pearson correlation; ^*∗∗*^Spearman correlation; CAEP: Cortical Auditory Evoked Potential; HF: high frequency (0,15 to 0,4).
